# Personalized E-Coaching in Cardiovascular Risk Reduction: A Randomized Controlled Trial

**DOI:** 10.5334/aogh.2496

**Published:** 2019-07-12

**Authors:** Mohammed Y. Khanji, Armida Balawon, Redha Boubertakh, Leonard Hofstra, Jagat Narula, Myriam Hunink, Francesca Pugliese, Steffen E. Petersen

**Affiliations:** 1 Centre for Advanced Cardiovascular Imaging and Research, William Harvey Research Institute, Queen Mary University of London, UK; 2 Barts Health NHS Trust, London, UK; 3 Cardiologie Centra Nederland, Utrecht, NL; 4 Mount Sinai Heart, Icahn School of Medicine at Mount Sinai, New York, US; 5 Department of Clinical Epidemiology and Radiology, Erasmus MC, Rotterdam, NL; 6 Center for Health Decision Sciences, Harvard T.H. Chan School of Public Health, Boston, MA, US

## Abstract

**Objectives:**

To assess whether electronic (e-) coaching, using personalized web-based lifestyle and risk factor counselling with additional email prompts, provides additional risk reduction when added to standard of care (SOC) in individuals at increased risk.

**Methods:**

Between June 2013 and May 2015, 402 participants were allocated 1:1 to e-coaching and SOC versus SOC. Participants free of manifest cardiovascular disease, with internet access, and a 10-year QRISK2 cardiovascular risk of ≥10% were enrolled. Change in oscillometric carotid-femoral pulse wave velocity (PWV) from baseline to six months was the primary endpoint. Secondary outcomes included change in blood pressure (BP), weight, and risk scores. Analysis was by intention to treat.

**Results:**

Mean (±SD) age was 65.5 (5.6) years with 37% females. Primary outcome data were available for 94%. There was no difference in PWV reductions between e-coaching and standard of care groups (–0.16 m/s vs. –0.25 m/s, 95% confidence interval –0.39 to 0.22, p = 0.56). There were no differences in the improvement between groups for BP, weight, Framingham, or QRISK2 scores. Pulse wave velocity change was more favorable in those with a higher level of education (p = 0.04), but was not associated with age, gender, presence of diabetes, baseline QRISK2 score, or logins to the website.

**Conclusions:**

In individuals at increased cardiovascular risk, a comprehensive ‘health check’ program modestly reduced future risk. Personalized e-coaching did not provide added risk reduction. Currently there is no evidence to routinely recommend e-coaching in cardiovascular health check programs.

**Trial registration:**

HAPPY London ClinicalTrials.gov: NCT01911910

## Introduction

The rise in obesity, diabetes and an aging population raises concern for future incidence and prevalence of cardiovascular disease (CVD). Primary prevention of CVD through risk factor reduction may reduce future burden [1,2,3]. Innovative measures to improve cardiovascular health are needed with nine potentially modifiable risk factors accounting for over 90% of the population attributable risk of a first myocardial infarction based on the findings from the INTERHEART Study [4]. Cochrane systematic reviews have concluded that interventions using counselling and education aimed at behavior change do not reduce mortality or clinical events in the general population but may be effective in reducing mortality in high risk populations [5]. There is some evidence that behavior change using computer tailoring can be effective in changing lifestyle and risk factors [6,7,8]. Internet and email access have increased globally and electronic (e-) coaching may aid preventative strategies, potentially allowing efficient, easy to use and cost-effective ways to improve the health and wellbeing of many. E-coaching may encourage individuals to improve lifestyle factors through identifying individual needs, setting personalized goals, using strategies to support change and reinforcement during the process [6]. This may be possible without overburdening existing healthcare facilities and encourage self-care and autonomy in managing personal health. The use of e-coaching has been applied to single risk factor modification with variable but mostly positive findings with dietary behavior change [9], increasing physical activity [10], and smoking cessation [11]. Though no differences were seen when addressing multiple risk factors in patients with familial hypercholesterolemia [12].

The Heart Attack Prevention Program for You (HAPPY) initially provided generic lifestyle e-coaching in a Dutch population over 3 months [13]. Participants with intermediate to high CVD risk (n = 141) were followed over 12 months with a relative reduction in CVD risk of 13.8% using the PROCAM risk score. The study did not have a control group and was not randomized.

## Aim

To assess the clinical effectiveness of personalized e-coaching as a primary prevention tool to reduce CVD risk in individuals with increased 10-year CVD risk.

## Methods

### Study design

The HAPPY London trial was a single-center, two-arm randomized controlled trial with 1:1 allocation to e-coaching and SOC versus SOC alone, stratified into moderate (QRISK2 10–20%) and high-risk (QRISK2 ≥ 20%), (ClinicalTrials.gov: NCT01911910). From June 2013 to November 2014, 402 adults were recruited based on guideline recommended risk scores to identify individuals at increased risk of developing CVD to facilitate risk reduction [14,15,16]. Follow-up was for 6 months with a final visit in May 2015. The study was not blinded.

### Participants

Participants were aged between 40 and 74 years and had a 10-year CVD risk score of 10% or higher based on the UK validated QRISK2 score [17]. They needed to have easy access (i.e. home computer, mobile phone, local library) to the Internet and email and have sufficient fluency of the English language to understand and comply with the written and verbal advice. Participants were excluded if they had established CVD (myocardial infarction, stroke or angina) or life-threatening conditions.

Recruitment was primarily by postal invitation for potentially eligible individuals identified from primary care database searches. Potential participants completed a registration and screening questionnaire on the study website (www.happylondon.info) to check eligibility. Screening visit was booked using online scheduling. Three subsequent visits took place at the William Harvey Heart Centre; baseline (within 2 weeks of screening visit), 3-months and 6-months from baseline. Randomization occurred prior to the baseline visit. Email appointment reminders were sent prior to visits. Assessments included lifestyle (including detailed dietary intake) and quality of life questionnaires (EQ-5D-3L, SF-36, recent physical activity questionnaire [RPAQ]), blood pressure (BP, Omron 705IT, Omron Corporation, Kyoto, Japan), blood tests, following an 8-hour fast (lipid profile, glucose, high sensitive C-reactive protein [hsCRP] and estimated glomerular filtration rate [eGFR]), carotid ultrasound scan (Panasonic Cardio Health System, Panasonic Healthcare Co. Ltd, Yokohama, Japan) and oscillometric method to assess PWV and pulse wave analysis (Vicorder device, Skidmore Medical, UK).

All participants gave written informed consent. The study was approved by the national Research Ethics Committee (NRES committee – London, REC reference 13/LO/0094) and was conducted in accordance with the declaration of Helsinki.

### Patient and Public Involvement

Patients and public were involved during trial design including assessing feasibility of taking part in a lifestyle intervention trial with requirement for internet access, reviewing and providing feedback on the patient information sheet, consent form, and advertisement posters.

### Randomization

Randomization was performed using an in-house software tool with allocation sequence concealment using two strata. Randomization into the treatment or control group was performed after confirming eligibility (see supplementary material for more details).

### Intervention

E-coaching involved computer-tailoring, a method of assessing individuals’ suboptimal lifestyle and risk factors and selecting communication content using data-driven decision rules that produce feedback automatically from a database of content options. Participants had personal logins and passwords. Written information was provided on the website with links to other sites and online videos. The HAPPY London web-based tool provided a personalized score for their lifestyle and 10-year CVD risk score, provided tailored advice and information specifically for those suboptimal factors that were relevant to that participants. Ideal targets were highlighted as goals and then updated following 3 and 6 months visits, providing dynamic tailoring to increase efficacy [6]. Dynamic tailoring entailed updating the participants lifestyle and risk factor profiles and personalized advice based on the follow-up assessments. Additional regular email reminders were sent to encourage achievement of goals. The email reminders were correlated to the number of suboptimal factors. Participants received instructions on how to use the website, lasting 5–10 minutes during the baseline visit. The web team (web developers and programmers) developed the content with input from the research team members (MYK and SEP).

### Standard of Care

All participants received personalized face-to-face counselling on suboptimal lifestyle and cardiovascular risk factors based on guideline recommendations once during the baseline visit, lasting 10–15 minutes [15]. All participants completed a lifestyle questionnaire. Advice on factors including blood pressure, cholesterol, glucose readings, smoking, weight, physical activity, fruit, and vegetable intake, alcohol intake and stress was provided by a trained physician (MYK).

### Outcomes

Primary outcome was baseline to 6-months PWV change. Secondary outcomes included changes in BP, weight, cholesterol, glucose, hsCRP, carotid intima media thickness, quality of life, Framingham risk score, QRISK2 scores, and self-reported physical activity using the validated RPAQ.

### Pulse Wave Velocity

Carotid femoral PWV and augmentation index, markers of global arterial stiffness, were measured using the Vicorder device. Measurements were obtained by placing a 10cm wide blood pressure cuff around the upper left thigh measuring the femoral pulse and a 3cm partial cuff around the neck at the level of the left carotid artery. The path-length was calculated according to manufacturer’s instructions, from suprasternal notch to a defined point on the upper part of the femoral cuff. The cuffs were inflated simultaneously to 65 mmHg with 2 high quality waveforms simultaneously recorded for 3 seconds using a volume displacement method. The foot-to-foot transit time was measured and values for carotid-femoral PWV were derived automatically by the software (Supplementary Figure 1). The average of 2 PWV measures was used.

### Cardiovascular risk calculation

A 10-year QRISK2 risk score was calculated once blood results were available at baseline, three-month, and six-month visits. Due to changes in the QRISK2 algorithm during the study period we recalculated this for all visits at the end of the study using the most up-to-date QRISK2 (https://qrisk.org) algorithm for standardization.

### Sample size calculation

Sample size calculation was based on a two-sample t-test with equal variances. The Type I error was set at 5% (two-sided). The standard deviation of 0.29 m/s for Vicorder measured PWV were based on published inter-study reproducibility data [18]. We proposed a sample size of 200 patients in each treatment arm assuming a dropout rate of 15–20% at the follow-up visit and having 80% power to detect a 0.1 m/s difference in PWV between the treatment groups (Supplementary Table 1).

### Statistical analysis

Analysis was performed on an intention to treat basis using R statistical software (version 3.4.4). Mean ± standard deviation was used for normally distributed variables. Median and interquartile ranges were used for data not normally distributed. T-test was used for normally distributed continuous measures and chi-square for categorical variables. Where variables were not normally distributed non-parametric tests were used (Wilcoxon rank sum test). Six-month changes in PWV and other parameters between treatment and control arms were compared for statistical difference. Agreement between repeated measurements was analyzed using student’s paired t-test. Reproducibility was assessed with intraclass correlation coefficient and Bland-Altman Plots. Regression analysis was used to assess for potential covariates that predicted PWV change.

## Results

**Figure 1:**
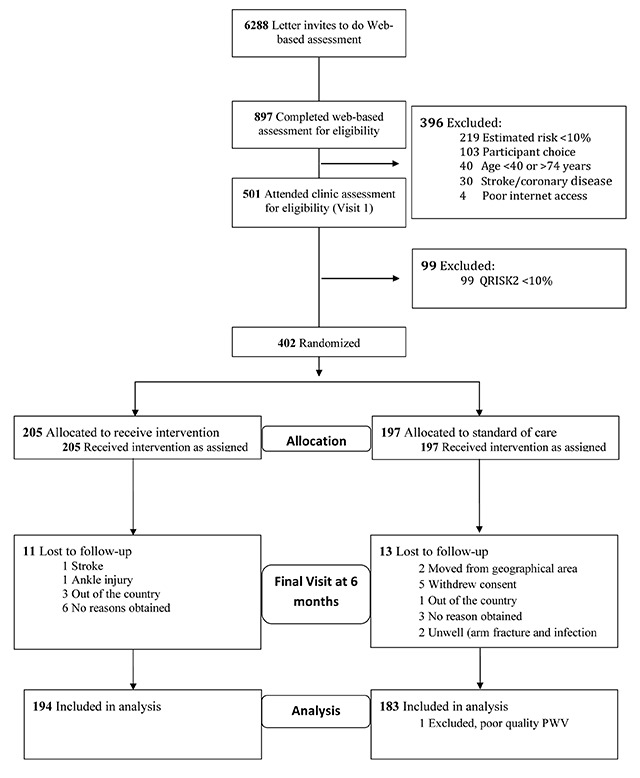
CONSORT flow diagram for the HAPPY London study.

The online ‘mini-check’ for eligibility was completed by 891 people and 501 fulfilled the preliminary inclusion criteria and accepted invitation for participation. Of these, 402 had a QRISK2 score of 10% or more and were randomized (see Figure 1, CONSORT flow diagram). Mean age was 65.5 ± 5.6 years and 37% were females. Median 10-year QRISK2 score was 16.5% and mean Framingham Risk score was 17.5%. Demographics and baseline characteristics including CVD risk factors are included in Table 1. The proportion of participants with a high QRISK2 score was similar in both the e-coaching and SOC groups (36% vs. 31%). During the study there were no important harms or unintended effects experienced by the participants (see Supplementary Table 2 for full study CONSORT checklist).

**Table 1:** Demographics and baseline clinical characteristics for both groups.

Clinical Values	E-coaching group (n = 205)	SOC group (n = 197)	P value

Age (years)	65.1(6.3)	65.9(4.8)	0.15
Male sex, no. (%)	127(62.0)	126(64.0)	0.75
University education, no. (%)	109(53)	97(49)	0.42
Smoking history, no. (%)			
Non-smoker	104(50.7)	80(40.6)	0.053
Ex-smoker	86(42.0)	101(51.3)	0.08
Light (<10 cigarettes/day)	9(4.4)	9(4.6)	1
Moderate (11–19)	6(2.9)	3(1.5)	0.53
Heavy (>20)	0	4(2.0)	0.12
Ethnicity, no. (%)			
White	182(88.8)	172(87.3)	0.76^§^
Asian	20(9.8)	14(7.1)	0.76^§^
Black Afro-Caribbean	1(0.5)	5(2.5)	0.76^§^
Other	2(1.0)	6(3.0)	0.76^§^
Medical History			
Rheumatoid Arthritis, no. (%)	8(3.9)	3(1.5)	0.25
BP medication, no. (%)	105(51.2)	83(42.1)	0.08
Cholesterol medication, no. (%)	98(47.8)	84(42.6)	0.40
Diabetes, no. (%)	35(17.1)	22(11.2)	0.12
Atrial fibrillation, no. (%)	9(4.4)	11(5.6)	0.75
Family History CAD, no. (%)	75(36.6)	60(31)	0.25
Risk Scores			
QRISK2*, 10-year risk, %	16.5(12.7; 23.1)	16.5(12.6; 21.1)	0.56
Framingham risk score, 10-year risk, %	16(9)	17.8(10)	0.54
Risk Stratification			
High risk, no. (%)	73(36)	62(31)	0.38
QRISK2 for high risk*, 10-year risk, %	25.7(22.2; 31.2)	26.3(21.8; 31.2)	0.99
Mod risk (n)	132(64)	135(69)	0.38
QRISK2 for mod*, 10-year risk, %	13.9(11.8; 16.2)	13.7(11.6; 16.7)	0.95
High risk or diabetes, no. (%)	79(39)	67(34)	0.4
Systolic BP, mmHg	132.5(13.3)	132.3(14.8)	0.88
Diastolic BP, mmHg	79.2(9.2)	80(8.6)	0.34
Weight, Kg	80.7(18.4)	79.7(16)	0.56
BMI, Kg/m^2^	28.1(5.6)	27.4(4.4)	0.16
Hip circumference, cm	104.7(10.3)	103.6(8.2)	0.25
Waist circumference, cm	95.8(15.2)	95.4(12)	0.81
Total Cholesterol, mmol/L	4.9(1.1)	5.1(1.1)	0.09
HDL, mmol/L	1.6(0.5)	1.6(0.4)	0.95
LDL, mmol/L	2.8(1)	2.9(1)	0.10
Triglyceride*, mmol/L	1.1(0.8; 1.5)	1.3(0.8; 1.5)	0.31
Glucose*, mmol/L	5.5(5.1; 6.0)	5.5(5.1; 5.9)	0.71
hsCRP*, mg/L	1.2(0.7; 2.5)	1.3(0.7; 2.4)	0.76
eGFR, mL/min/1.73 sqm	82.9(20.1)	81.9(17.9)	0.58
Physical activity, minutes per day	70.8(75.6)	64.9(91.9)	0.48
Lifestyle score, out of 10, 10 being best score	6.9(1.3)	6.8(1.3)	0.22
**Surrogate Markers**			

PWV, m/s	8.5(1.7)	8.9(1.6)	0.027*
CIMT (combined right and left), mm	0.705(0.13)	0.736(0.13)	0.02*
Quality of Life			
Self-rated health state*, best health score 100	80(65–90)	80(65–90)	0.21
EQ-5D-3L VAS value*, best quality of life score 1)	0.76(0.73; 01.0)	0.76(0.69; 1.0)	0.39

Results presented as mean (SD) or *median (interquartile range), unless stated otherwise. ^§^ white vs. non-white. Abbreviations: BMI, body mass index; BP, blood pressure; CAD, coronary artery disease; PWV, carotid-femoral pulse wave velocity; CIMT, carotid intima-media thickness; eGFR, estimated glomerular filtration rate; EQ-5D-3L VAS, Euroqol, 5 dimension, 3 level visual analogue scale – validated questionnaire; HDL, high-density lipoprotein cholesterol; hsCRP, high sensitivity C-reactive protein; IQR, inter quartile range; LDL, low-density lipoprotein cholesterol; Mod, moderate; pd, per day; QOL, quality of life.

The only reason for not being randomized into the study was having a 10-year QRISK2 score of less than 10%. During the study 24 participants (6%) dropped out and did not have follow-up measurements (11 from e-coaching group and 13 from the SOC). Following baseline assessment, statin medication was initiated in 12 versus 8 individuals and antihypertensive medication was initiated in 4 versus 4 individuals in the e-coaching and SOC group, respectively, by their primary care physician.

**Figure 2:**
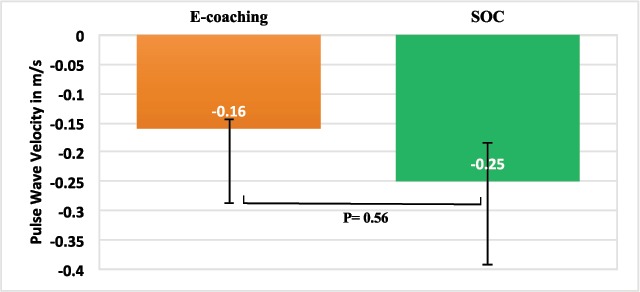
Change in carotid-femoral pulse wave velocity over six months.

After 6 months there was no significant difference in the reduction in the PWV between the e-coaching (–0.16 m/s) and SOC groups (–0.25 m/s), mean difference between groups 0.09 m/s, 95 % confidence interval (CI) –0.39 to 0.22, p = 0.56, Cohen’s d = 0.05 (Figure 2). Even when baseline difference was adjusted, there remained no difference in the 6-month PWV between the e-coaching and SOC groups –0.017 m/s vs –0.025 m/s, CI –0.029; 0.044, p = 0.679.

Table 2 summarizes the changes seen at follow up in each group. There were similar modest improvements seen between the e-coaching and SOC, including improved systolic BP, diastolic BP, weight, hip circumference, waist circumference, fasting total cholesterol, fasting LDL cholesterol, fasting triglyceride, fasting glucose, and a reduction in the 10-year Framingham Risk score (see Supplementary Table 3 for baseline and six-month differences in each group and Supplementary Figure 2 for trends in changes from baseline and the three-month and six-month visits). Lifestyle improvements were also seen in both groups, including a reduction in alcohol intake, increased moderate physical activity, measured as minutes per week averaged over a five-day week (25 minutes vs. 8 minutes, p = 0.54), and an improved overall lifestyle score which considered a number of lifestyle factors, with the best possible score of 10 (0.74 vs. 0.66, p = 0.45). There was also an improvement in the proportion of participants achieving guideline-based targets for risk factors including systolic BP, diastolic BP, BMI, cholesterol levels, waist circumference and achieving over 150 minutes moderate physical activity per week which was similar in both groups (Supplementary Table 4).

**Table 2:** Comparison of change over six months between intervention group and SOC group.

Clinical Values	E-coaching group n = 194		SOC group n = 183		Mean difference between groups over six months (95% CI)	p-value

	Follow-up	Mean change over 6 months	Follow-up	Mean change over 6 months		

Primary Endpoint						

PWV^†^,	**8.3 (1.4)**	**–0.16**	**8.6 (1.7)**	**–0.25**	**0.09 (–0.39; 0.22)**	**0.56**
Secondary Endpoints						

Systolic BP, mmHg	129.5 (13.6)	–3.18	130.7 (14.7)	–1.688	–1.5 (–4.2; 1.2)	0.27
Diastolic BP, mmHg	76.7 (9.1)	–2.37	78 (8.7)	–2.076	–0.29 (–1.6; 1.1)	0.67
Weight, kg	78.9 (18.1)	–1.22	79 (15.8)	–0.763	–0.45 (–1.0; 0.1)	0.10
BMI, kg/m2	27.4 (5.3)	–0.42	27.1 (4.4)	–0.247	–0.17 (–0.4; 0)	0.07
Hip circumference, cm	102.2 (9.9)	–2.19	101.8 (8.2)	–1.809	–0.38 (–1.2; 0.5)	0.37
Waist circumference, cm	92.7 (14.7)	–2.55	93.4 (12)	–2.048	–0.5 (–1.5; 0.5)	0.31
Total Cholesterol, mmol/L	4.8 (1)	–0.16	4.9 (1)	–0.197	0.04 (–0.1; 0.2)	0.60
HDL, mmol/L	1.6 (0.5)	–0.03	1.6 (0.4)	–0.017	–0.01 (–0.1; 0)	0.64
LDL, mmol/L	2.6 (0.9)	–0.10	2.8 (0.9)	–0.142	0.04 (–0.1; 0.2)	0.56
Triglyceride, mmol/L	1.2 (0.8)	–0.08	1.2 (0.6)	–0.113	0.03 (–0.1; 0.1)	0.56
Glucose, mmol/L	5.6 (1.3)	–0.29	5.5 (1.1)	–0.266	–0.03 (–0.2; 0.2)	0.77
HsCRP, mg/L	2.3 (5.9)	–0.26	2.2 (5.4)	0.004	–0.27 (–1.9; 1.3)	0.75
eGFR, mL/min/1.73sqm)	82.3 (17)	–0.64	83 (20.2)	1.225	–1.87 (–4.8; 1.1)	0.22
Alcohol per week, units	7.9 (9.4)	–0.78	7.7 (8.2)	–1.081	0.3 (–0.7; 1.3)	0.54
Physical activity, minutes per day	98 (145.5)	25.10	74.2 (100.3)	8.448	16.65 (–10.3; 43.6)	0.23
Lifestyle score, best score 10	7.7 (1.2)	0.74	7.5 (1.3)	0.659	0.08 (–0.1; 0.3)	0.45
CIMT (left and right), mm	0.72 (0.14)	0.014	0.75 (0.13)	0.015	0.02 (–0.017; 0.015)	0.91
QRISK2 score, 10-year risk, %	19.2 (8.5)	0.14	18.9 (8.6)	0.01	0.24 (–0.4; 0.6)	0.63
Expected QRISK2 at 6 months^#^, 10-year risk, %	19.6 (8.1)		19.3 (8.8)			
Framingham risk score, 10-year risk, %	16.1 (8.9)	–1.23	16.6 (9.5)	–1.37	0.14 (–0.9; 1.2)	0.79
Self-rated health state (best health 100)*	85 (75; 90)	4.81	84 (69; 90)	5.58	–0.77 (–6.5; 4.9)	0.79
EQ-5D-3L VAS value (best value 1)*	0.76 (0.7; 1.0)	–0.00	0.76 (0.70; 1.0)	0.01	–0.01 (–0.02; 0.05)	0.44
Total logins to study Website, n		12.8		9.1		<0.001

Results presented as mean (SD) or *median (interquartile range), unless stated otherwise. Abbreviations: BMI, body mass index; BP, blood pressure; CAD, coronary artery disease; CIMT, carotid intima-media thickness; eGFR, estimated glomerular filtration rate; EQ-5D-3L VAS, Euroqol, 5 dimension, 3 level visual analogue scale – validated questionnaire; HDL, high-density lipoprotein cholesterol; hsCRP, high sensitivity C-reactive protein; IQR, inter quartile range; LDL, low-density lipoprotein cholesterol; Mod, moderate; n, number; PWV, carotid-femoral pulse wave velocity; QOL, quality of life. ^#^ Naturally expected due to older age only if other factors unchanged. QRISK increases with increasing age. ^†^ Corrected when extreme outlier excluded (a single reading of 27 m/s which was very different to a subsequent visit for the same patient and compared to all other readings).

Self-rated health state scores improved for both groups. The sum of the visual analogue scale value, which provides an indexed value based on the combination of the 5 dimensions of the EQ-5D-3L questionnaire, did not show any change over the study period either within or between the groups. There was no reduction in mean carotid intima media thickness (CIMT) over 6-months in either group. The mean number logins to the HAPPY London website was 13 for the e-coaching group over the six-month study period.

Univariate analysis showed that a higher level of education was a predictor of more favorable change in PWV only in the e-coaching group (–0.37 (0.18), p = 0.04). Change in PWV was not associated with age, gender, presence of diabetes or hypertension, baseline QRISK2 score, or the total number of logins to the website in either the e-coaching or SOC groups.

The reproducibility of PWV was good. The difference between repeated PWV measurements was –0.14 +/– 0.5 m/s (p = 0.38) with limits of agreement of –1.10 to 0.82 m/s and intra-class correlation coefficient was 0.89 (p < 0.001).

## Discussion

To our knowledge this is the first study to assess the impact of personalized e-coaching in a high-risk primary prevention cohort using robust cardiovascular surrogate markers. This study showed that e-coaching, using Internet and email-based heart attack prevention program on top of the SOC, compared with SOC alone did not further reduce cardiovascular risk based on interval change of the PWV surrogate marker and other CVD risk markers. E-coaching and SOC both modestly improved PWV and a number of cardiovascular risk factors that translated in a reduced estimated 10-year CVD risk based on QRISK2 and Framingham scores. Similar to previous studies, the frequency of logins to the website was low in the e-coaching group suggesting low degree of engagement with the online content. Given previous studies showing similar low engagement with website information, we had pre-emptively provided personalized emails as encouragement and reminders and the option of nominating a ‘buddy’ to help with motivation, to complement the website content.

Higher level of education was associated with favorable reduction in PWV in the e-coaching group. The potential reasons for this may be linked to better understanding of the content and better means and ability to implement lifestyle modification.

### Impact of Electronic Coaching in Prevention

Previous outcomes of e-coaching studies have been varied and the end points, designs, and strategies utilized have been heterogeneous. Limited studies exist in primary CVD prevention. A meta-analysis assessing the effectiveness of Internet-based interventions targeting participants with increased CVD risk identified five randomized controlled trials in diabetic patients and four in those with increased CVD risk predominantly due to high BP. Authors noted shortage of studies investigating the effectiveness of Internet-based interventions on direct CVD outcomes such as cardiac mortality or adverse events. They noted some evidence suggesting that interactive self-management programs that include lifestyle education and self-monitoring of health behaviors may be of benefit in improving some clinical CVD risk factors such as BP [19]. However, most studies concentrated on patients with either diabetes or hypertension.

Studies assessing the impact of smart phones and wearable mobile technology in CVD risk reduction are limited and, where available, often rely on self-reporting and do not conform to an intention to treat analysis [20]. A recent study in young adults with increased weight found that the addition of wearable technology to standard behavioral intervention resulted in less weight loss over 2 years suggesting that devices that monitor and provide feedback on physical activity may not offer an advantage over standard behavioral weight loss approaches [21].

Optimum follow-up period is debatable. Studies with follow-up of 6-months or less with weight loss have shown group differences [22]. Six-month follow-up was selected to capture sustainability of behavioral changes allowing time for detectable effects to take place. Longer follow-up beyond six months may have been informative for longer-term differences, although this seems unlikely to change the findings based on the 3- and 6-month trends showing convergence in the primary end point of PWV between the two groups (Supplementary Figure 2).

Primary prevention studies generally require long follow-up and large participant numbers to powerfully detect hard cardiac end points. This creates obvious resource and administrative challenges. Use of robust surrogate markers can help identify potentially useful interventions that can then be further studied. We therefore used PWV which is one of the most validated method to non-invasively measure arterial stiffness and is recognized as a surrogate marker for future cardiovascular events [23]. It is considered the gold standard index of aortic stiffness, as it is a relatively simple method with reported accuracy, reproducibility and an independent and strong predictor of adverse outcomes [24,25]. There is however only weak indirect evidence of change in PWV and long-term outcomes [26]. A study is under way to clarify this gap [27]. Finally, we only assessed the impact of adding e-coaching to SOC but did not test whether it could be effective when used as a part replacement to some SOC visits.

Our study included individuals who had high total cardiovascular risk. CVD risk assessment, using risk calculators to guide management, is now widely advocated in many primary prevention guidelines [14,15,16]. Our study utilized robust and sensitive surrogate markers, which confirmed that risk reduction programs have a modest improvement in future risk in general, but no additional clinical benefit with the addition of e-coaching. It may be argued that more information may provide motivation for future change or increase awareness of risk factors and health information. However, in resource limited healthcare systems rationalization occurs, with resources diverted from interventions that do not show objective benefit towards those with evidence of effectiveness.

### Strengths and Limitations

Our study is one of the largest of its kind and included a variety of sensitive measures to evaluate outcomes. The Internet tool and email reminders incorporated aspects of e-coaching that have shown benefit including personalization, dynamic feedback, goal setting and incorporation of social media. We had a very low drop-out rate of only 6%.

There were some limitations to our study. Researchers were not blinded and although we were strict in following the pre-specified protocol this may have led to possible bias. The adherence to the website segment of the e-coaching tool was low. We had anticipated this and added complementary features such as email reminders to overcome this known issue. Mobile phone applications and platform optimization may increase participant interaction with the e-coaching and may improve effectiveness. Volunteering effect may have been present and our cohort represented more Caucasians, males, and those with higher educational qualifications than the general London population, thus making generalizability challenging. Only people with Internet access were eligible which may have created a selection bias.

## Conclusions

In individuals at increased cardiovascular risk, a comprehensive ‘health check’ program modestly reduced future risk. Personalized e-coaching did not provide added risk reduction. Currently there is no evidence to routinely recommend e-coaching in cardiovascular health check programs.

## Transparency Declaration

The lead author affirms that this manuscript is an honest, accurate, and transparent account of the study being reported; that no important aspect of the study has been omitted; and that any discrepancies from the study as planned and registered have been explained.

## What is already known on this topic

Rising obesity, diabetes and aging population is likely to lead to increased incidence and prevalence of cardiovascular disease, which is already a leading cause of morbidity and mortality.

Interventions using counselling and education for behavior change do not reduce mortality or clinical events in the general population but may be effective in high risk population.

There is some evidence that electronic coaching can be effective in improving lifestyle and risk factors when addressing single risk factors.

## What this study adds

Face to face counselling for lifestyle and risk factors from a health care professional provides modest improvements in cardiovascular risk.

Electronic coaching, using personalized website and email reminders, does not provide additional risk reduction when added to standard of care in individuals considered at high risk. Although participants with higher levels of education may derive some benefit.

## How might this impact on clinical practice

We found no evidence to routinely recommend electronic coaching on top of current standard of care in primary prevention programs for individuals at increased risk. Digital and mobile health systems should undergo careful evaluation for effectiveness before being rolled out in prevention or health and wellbeing programs particularly given resource constraints.

## Additional Files

The additional files for this article can be found as follows:

10.5334/aogh.2496.s1 Supplementary Figure 1. Method for calculating the transit time (TT) used to derive carotid-femoral PWV, using the ‘foot-to-foot’ using the Vicorder device.

10.5334/aogh.2496.s2 Supplementary Figure 2. Change in parameters at three- and six-month follow-up.

10.5334/aogh.2496.s3 Online Only Supplement. CONSORT checklist, supplementary Tables and further study methods and summary details.

## Abbreviations

BP = Blood Pressure

CIMT = Carotid Intima Media Thickness

CVD = Cardiovascular Disease

E = Electronic

eGFR = estimated Glomerular Filtration Rate

HAPPY = Heart Attack Prevention Program for You

hsCRP = High sensitive C-reactive Protein

PWV = Pulse Wave Velocity

RPAQ = Recent Physical Activity Questionnaire

SOC = Standard of Care
