# Supplementary material for: Personalized E-Coaching in Cardiovascular Risk Reduction: A Randomized Controlled Trial
Source: Ann Glob Health. 2019 Jul 12;85(1):107. doi: 10.5334/aogh.2496 (PMC6634325; doi:10.5334/aogh.2496)

Supplementary Figure 1. Method for calculating the transit time (TT) used to derive carotid-femoral PWV, using the 'foot-to-foot' using the Vicorder device.

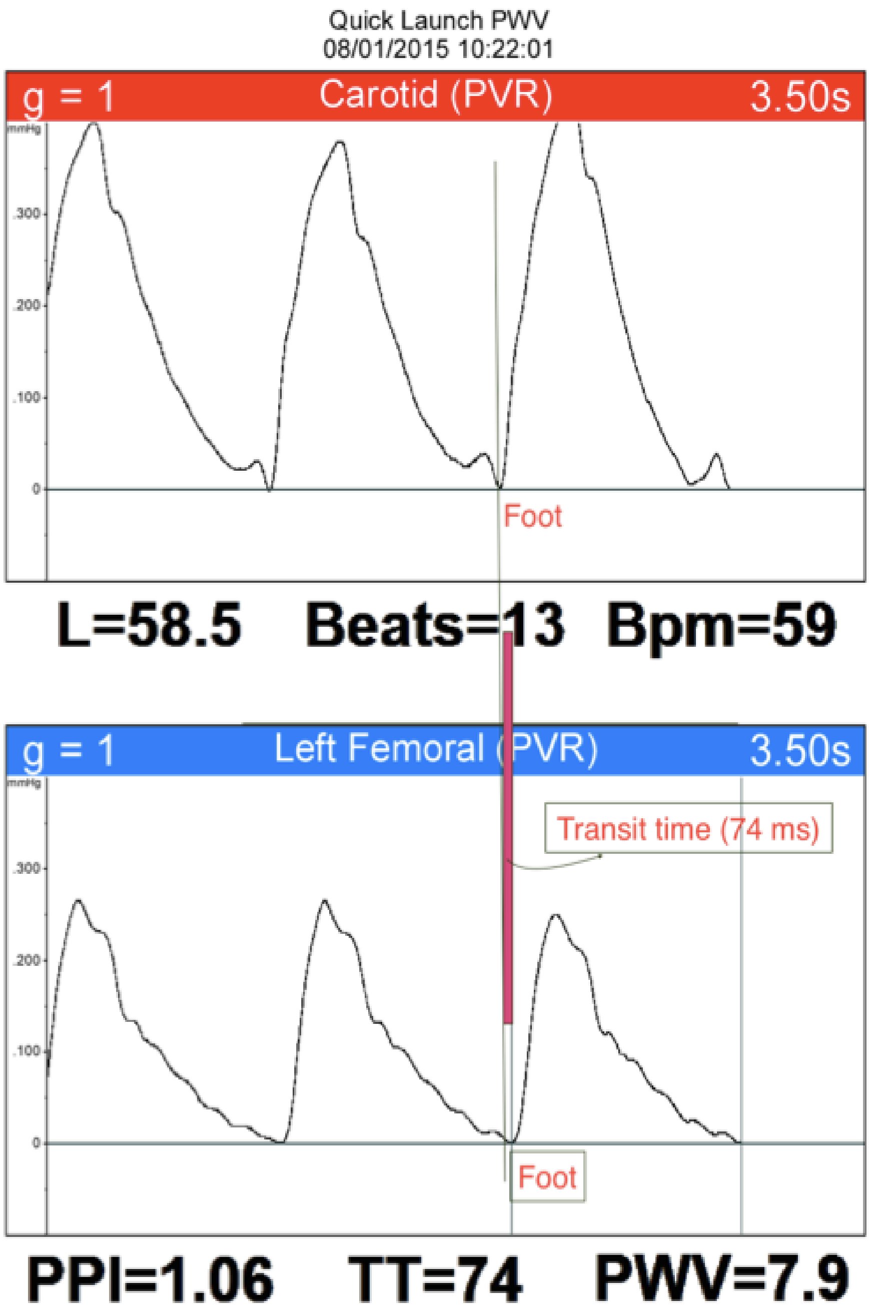

Supplement: Supplementary Figure 1. — Method for calculating the transit time (TT) used to derive carotid-femoral PWV, using the ‘foot-to-foot’ using the Vicorder device. [file agh-85-1-2496-s1.pdf]
