# Supplementary figures and images for: Personalized E-Coaching in Cardiovascular Risk Reduction: A Randomized Controlled Trial
Source: Ann Glob Health. 2019 Jul 12;85(1):107. doi: 10.5334/aogh.2496 (PMC6634325; doi:10.5334/aogh.2496)

## Changes in parameters at 3-month and 6-month follow-up

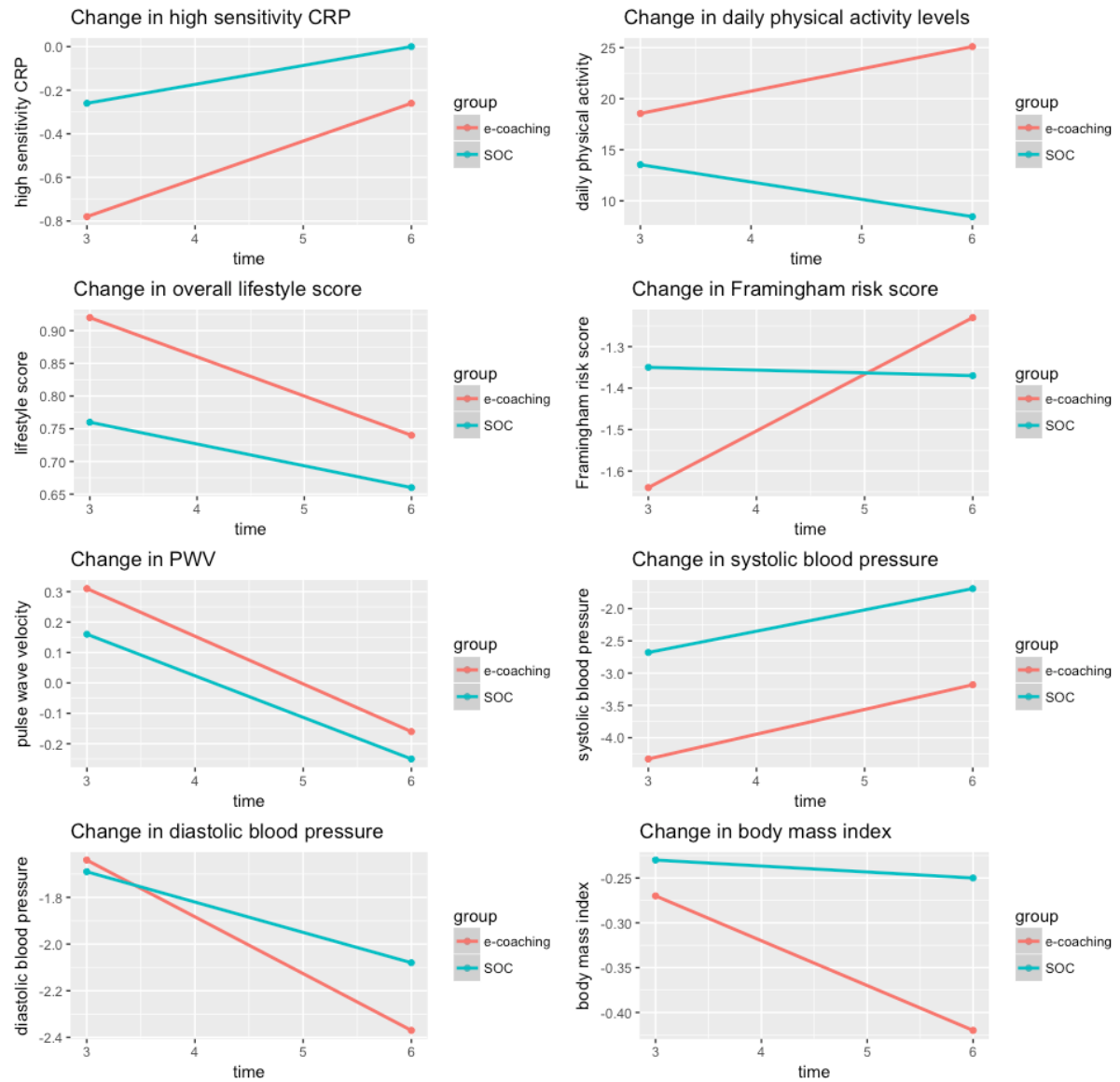

Supplement: Supplementary Figure 2. — Change in parameters at three- and six-month follow-up. [file agh-85-1-2496-s2.pdf]
