# Supplementary material for: Personalized E-Coaching in Cardiovascular Risk Reduction: A Randomized Controlled Trial
Source: Ann Glob Health. 2019 Jul 12;85(1):107. doi: 10.5334/aogh.2496 (PMC6634325; doi:10.5334/aogh.2496)
Supplement: Online Only Supplement. — CONSORT checklist, supplementary Tables and further study methods and summary details. [file agh-85-1-2496-s3.pdf]

Online Only Supplement

Supplementary Table 1. Sample size calculations with sensitivity analysis for PWV

**Type 1 error 5%**	**95% power**	**90% power**	**80% power**
0.1 m/s	220	178	133
0.15 m/s	99	80	60
0.2 m/s	56	46	34
0.25 m/s	36	30	23

Type I error, 5% (two-sided), two-sample t-test with equal variances, standard deviations of difference of 0.29 m/s for pulse wave velocity.

Supplementary Table 2: CONSORT checklist

Section/Topic	Item No	Checklist item	Reported on page No
Title and abstract			
	1a	Identification as a randomized trial in the title	1
	1b	Structured summary of trial design, methods, results, and conclusions (for specific guidance see CONSORT for abstracts)	3
Introduction			
Background and objectives	2a	Scientific background and explanation of rationale	4
	2b	Specific objectives or hypotheses	5
Methods			
Trial design	3a	Description of trial design (such as parallel, factorial) including allocation ratio	5
	3b	Important changes to methods after trial commencement (such as eligibility criteria), with reasons Addition of PFT for additional analysis	8
Participants	4a	Eligibility criteria for participants	6 and supplement
	4b	Settings and locations where the data were collected	7 and supplement
Interventions	5	The interventions for each group with sufficient details to allow replication, including how and when they were actually administered Interventions were performed face to face during the baseline visit. During this visit those in the e-coaching arm also received instructions on how to use the website and what to expect regards emails.	7 and supplement
Outcomes	6a	Completely defined pre-specified primary and secondary outcome measures, including how and when they were assessed	8 and 9
	6b	Any changes to trial outcomes after the trial commenced, with reasons	N/A
Sample size	7a	How sample size was determined	9, 10 and supplement
	7b	When applicable, explanation of any interim analyses and stopping guidelines	N/A
Randomization:			
Sequence generation	8a	Method used to generate the random allocation sequence	7 and supplement
	8b	Type of randomization; details of any restriction (such as blocking and block size)	7 and supplement
Allocation concealment mechanism	9	Mechanism used to implement the random allocation sequence (such as sequentially numbered containers), describing any steps taken to conceal the sequence until interventions were assigned	7 and supplement
Implementation	10	Who generated the random allocation sequence, who enrolled participants, and who assigned participants to interventions	7 and supplement
Blinding	11a	If done, who was blinded after assignment to interventions (for example, participants, care providers, those assessing outcomes) and how	N/A
	11b	If relevant, description of the similarity of interventions	7,8
Statistical methods	12a	Statistical methods used to compare groups for primary and secondary outcomes	10
	12b	Methods for additional analyses, such as subgroup analyses and adjusted analyses	N/A

Results			
Participant flow (a diagram is strongly recommended)	13a	For each group, the numbers of participants who were randomly assigned, received intended treatment, and were analysed for the primary outcome	10, CONSORT flow and Table 1
	13b	For each group, losses and exclusions after randomization, together with reasons	10 and CONSORT flow
Recruitment	14a	Dates defining the periods of recruitment and follow-up	6
	14b	Why the trial ended or was stopped	6
Baseline data	15	A table showing baseline demographic and clinical characteristics for each group	22
Numbers analysed	16	For each group, number of participants (denominator) included in each analysis and whether the analysis was by original assigned groups	10, Table 1 and CONSORT flow diagram
Outcomes and estimation	17a	For each primary and secondary outcome, results for each group, and the estimated effect size and its precision (such as 95% confidence interval)	27, 28 (Table 2)
	17b	For binary outcomes, presentation of both absolute and relative effect sizes is recommended	N/A
Ancillary analyses	18	Results of any other analyses performed, including subgroup analyses and adjusted analyses, distinguishing pre-specified from exploratory	N/A
Harms	19	All important harms or unintended effects in each group (for specific guidance see CONSORT for harms)<br>No harms	12 (None occurred)
Discussion			
Limitations	20	Trial limitations, addressing sources of potential bias, imprecision, and, if relevant, multiplicity of analyses	14 and 15
Generalizability	21	Generalizability (external validity, applicability) of the trial findings	114 and 15
Interpretation	22	Interpretation consistent with results, balancing benefits and harms, and considering other relevant evidence	15
Other information			
Registration	23	Registration number and name of trial registry	2
Protocol	24	Where the full trial protocol can be accessed, if available	2 and supplement
Funding	25	Sources of funding and other support (such as supply of drugs), role of funders	16

Supplementary Table 3

E-coaching group	Mean baseline n= 205	Mean 6 months n= 194	P value	mean of the differences	95% CI lower	95% CI upper
**Systolic BP, mmHg**	132.3	130.7	0.102	1.69	-0.34	3.71
**Diastolic BP, mmHg**	80.0	78.0	<0.001*	2.08	1.08	3.07
**Weight, kg**	79.7	79.0	<0.001*	0.76	0.39	1.14
**BMI, kg/m2**	27.4	27.1	<0.001*	0.25	0.12	0.38
**Hip circumference, cm**	103.6	101.8	<0.001*	1.81	1.19	2.43
**Waist circumference, cm**	95.4	93.4	<0.001*	2.05	1.35	2.75
**Total Cholesterol, mmol/L**	5.1	4.9	0.001*	0.2	0.09	0.31
**HDL, mmol/L**	1.6	1.6	0.343	0.02	-0.02	0.05
**LDL, mmol/L**	2.9	2.8	0.010*	0.14	0.03	0.25
**Triglyceride, mmol/L**	1.3	1.2	0.013*	0.11	0.02	0.2
**Glucose, mmol/L**	5.7	5.5	<0.001*	0.27	0.14	0.39
**hsCRP, mg/L**	2.2	2.2	0.994	0	-1.07	1.06
**eGFR, mL/min/ 1.73sqm)**	81.9	83.0	0.266	-1.23	-3.39	0.94
**Alcohol per week, units**	8.9	7.7	0.004*	1.08	0.34	1.82
**Physical activity, minutes per day**	64.9	74.2	0.353	-8.45	-26.34	9.44
**Lifestyle score, best score 10**	6.8	7.5	<0.001*	-0.66	-0.81	-0.51
**QRISK2 score, 10-year risk, %**	18.8	18.9	0.81	0.05	-0.34	0.43
**Framingham risk score, 10-year risk, %**	17.8	16.6	0.001*	1.37	0.54	2.2
**PWV**	**8.9**	**8.6**	**0.055**	**0.25**	**-0.01**	**0.5**

SOC group	Mean baseline n= 197	Mean 6 months n= 183	P value	mean of the differences	95% CI lower	95% CI upper
**Systolic BP, mmHg**	132.5	129.5	<0.001*	3.18	1.46	4.91
**Diastolic BP, mmHg**	79.2	76.7	<0.001*	2.37	1.46	3.28
**Weight, kg**	80.7	78.9	<0.001*	1.22	0.82	1.61
**BMI, kg/m2**	28.1	27.4	<0.001*	0.42	0.28	0.56
**Hip circumference, cm**	104.7	102.2	<0.001*	2.19	1.64	2.74
**Waist circumference, cm**	95.8	92.7	<0.001*	2.54	1.88	3.21
**Total Cholesterol, mmol/L**	4.9	4.8	0.001*	0.16	0.06	0.25
**HDL, mmol/L**	1.6	1.6	0.027*	0.03	0	0.05
**LDL, mmol/L**	2.8	2.6	0.020*	0.1	0.02	0.19
**Triglyceride, mmol/L**	1.3	1.2	0.004*	0.08	0.03	0.14
**Glucose, mmol/L**	5.8	5.6	<0.001*	0.29	0.17	0.42
**hsCRP, mg/L**	2.6	2.3	0.67	0.26	-0.95	1.47
**eGFR, mL/min/ 1.73sqm)**	82.9	82.3	0.534	0.64	-1.39	2.68
**Alcohol per week, units**	8.9	7.9	0.021*	0.78	0.12	1.43
**Physical activity, minutes per day**	70.8	98.0	0.016*	-25.1	-45.4	-4.8
**Lifestyle score, best score 10**	6.9	7.7	<0.001*	-0.74	-0.89	-0.59
**QRISK2 score, 10-year risk, %**	19.1	19.2	0.279	-0.2	-0.55	0.16
**Framingham risk score, 10-year risk, %**	17.2	16.1	<0.001*	1.23	0.6	1.86
**PWV**	**8.5**	**8.3**	**0.081**	**0.16**	**-0.02**	**0.33**

Abbreviations: BMI, body mass index; BP, blood pressure; eGFR, estimated glomerular filtration rate; HDL, high-density lipoprotein cholesterol; hsCRP, high sensitivity C-reactive protein; LDL, low-density lipoprotein cholesterol; n, number; PWV, carotid-femoral pulse wave velocity.

Supplementary Table 4. Proportions achieving target levels

	E-coaching group		SOC group		Comparison between groups
**Risk factors/markers**	Baseline	Follow-up	Baseline	Follow-up	Follow-up
	n=205	n=194	n=197	n=184	P value
**Systolic BP (<140mmHg)**	156 (76)	158 (81)	142 (72)	142 (77)	0.31
**Diastolic BP (<90 mmHg)**	176 (86)	178 (92)	172 (87)	164 (89)	0.39
**BMI (<25 kg/m2)**	65 (32)	74 (38)	62 (31)	66 (36)	0.65
**Total Cholesterol (<5 mmol/L)**	110 (54)	116 (60)	95 (48)	101 (55)	0.37
**LDL (<3 mmol/L)**	118 (58)	121 (63)	104 (53)	108 (60)	0.51
**Triglyceride (<1.7 mmol/L)**	164 (80)	170 (88)	159 (81)	154 (84)	0.33
**Glucose (<6.1 mmol/L)**	162 (79)	162 (84)	155 (79)	149 (81)	0.60
**Waist circumference***	117 (57)	127 (65)	120 (61)	119 (65)	0.87
**Physical activity (>150 mins/week)**	131 (64)	109 (65)	111 (57)	104 (60)	0.36

Abbreviations: BMI = Body mass index, BP = Blood pressure, E-coaching = Electronic coaching, LDL = low-density lipoprotein, SOC = Standard of care.
*<102cm males and <88cm females

Supplementary Table 5. Predictors of change in pulse wave velocity.

Variable	Pulse wave velocity m/s					
	Univariate<br><br>Whole group		Univariate<br><br>E-coaching		Univariate<br><br>SOC	
	B (se)	P-value	B (se)	P-value	B (se)	P-value
**Age, years**	-0.02 (0.01)	0.108	-0.02 (0.02)	0.243	-0.03 (0.03)	0.247
**Gender, female**	0.09 (0.16)	0.568	0.19 (0.18)	0.300	-0.02 (0.27)	0.933
**Education, university level**	-0.24 (0.16)	0.121	-0.37 (0.18)	0.040*	-0.11 (0.26)	0.668
**Diabetes**	0.26 (0.22)	0.243	0.31 (0.24)	0.201	0.17 (0.40)	0.676
**Hypertension**	0.05 (0.16)	0.751	0.07 (0.18)	0.705	0.01 (0.26)	0.961
**QRISK2, %**	0.00 (0.01)	0.754	-0.00 (0.01)	0.858	0.01 (0.02)	0.624
**Total logins to website**	-0.01 (0.01)	0.273	-0.01 (0.01)	0.425	-0.02 (0.02)	0.282

* $p<0.05$

Detailed protocol information
(Cardiovascular magnetic resonance subgroup protocol not included as will be presented in future subgroup analysis).

Inclusion criteria

Participants were deemed eligible for inclusion in this study if all of the following criteria were met:
1. Informed consent given by participant (see Appendix for copy of consent form)
2. Between 40 and 74 years of age
3. Had unrestricted access to the Internet
4. Were sufficiently fluent in English language (as judged by the research team). The subject had to be able to understand and comply with protocol requirements, instructions and protocol-stated restrictions
5. An estimated intermediate to high risk for CVD events based on the web-based pre-screening tool "mini-check" (www.happylondon.info), which is based on the non-laboratory FRS (≥10% 10-year CVD risk)
6. Following screening visit participants had to have a 10-year QRISK2 score of ≥10%.

Exclusion criteria

A subject was not eligible for inclusion in this study if they met any of the following criteria:

1. History of myocardial infarction
2. History of stroke or transient ischaemic attack
3. Cardiac sounding chest pain requiring further investigations
4. Current life-threatening conditions other than vascular disease (e.g. very severe chronic airways disease, human immunodeficiency virus positive, life-threatening arrhythmias) that may prevent a subject from completing the study
5. Only for subgroup undergoing cardiac contrast-enhanced magnetic resonance studies: Any contraindication to a contrast-enhanced magnetic resonance study, such as known allergies to gadolinium-based contrast agents, severe claustrophobia, pacemakers, defibrillators.

We also advertised through posters inside London buses, email invitations to university and hospital staff. All interested individuals had to register on the study website (www.happylondon.info) and complete a brief online 'mini-check' screening questionnaire to check for eligibility. 'Mini-check' eligible individuals who had an estimated 10-year cardiovascular risk score of above 10% were invited to book an appointment, using the online scheduler, to attend a screening visit at the research centre. Confirmation emails were sent with the appointment details and included the patient information sheet and a copy of the consent form.

Assessment and Follow up

'Mini-check' - Prior to first visit

Potential participants expressing an interest in this study were directed to the www.happylondon.info website for further information and registration. The CVD risk profile was estimated using a web-based tool ("mini-check"). The purpose of this step was to reduce screening visits. Thus, reducing potential cost and other resources required for the study, such as staff and research centre time. In the process I filtered out those who were not eligible due to having too low a risk or not meeting other eligibility criteria. It also allowed us to invite only those that had Internet access.
The mini-check consisted of questions regarding potential exclusion criteria (previous diagnosis of myocardial infarction, stroke or angina). They also had to tick "yes" to a question asking if they had easy access to the Internet. The non-laboratory Framingham Risk Score was calculated based on self-reported age, gender, history of diabetes, hypertension treatment, participant's estimated height and weight and a family history of premature CVD.

If the non-laboratory Framingham 10-year risk scores estimate was greater than or equal to 10% and there were no exclusion criteria, the participant was offered the opportunity to book an appointment for a physical examination using the online booking calendar. If the participants did not want to proceed with the study, a message advised them to discuss the result with their GP, if they were not previously

aware that they may have potentially increased risk of developing CVD in the next 10 years. This was in line with the NHS Health check recommendations. Those who had a score of less than 10% were informed that they were not eligible to enter the study.

From this pre-screened population we invited participants with an estimated mini-check 10-year risk score of 10% or more for CVD events, to attend the research centre to assess the actual CVD risk. They were sent an email confirming their chosen appointment slot along with the patient information sheet (PIS) and the consent form that they would sign during their chosen visit appointment (both forms in the Appendix section). Participants who were potentially eligible for the CMR scan also received a CMR safety questionnaire to review.

Screening visit

The screening visit took place at the Heart Centre, William Harvey Research Institute, Queen Mary University London and lasted approximately 35 minutes. As the visit required the participant to fast for the blood test, visits were limited to the morning time usually starting at 9am and the last slot at 12pm. If participants specifically requested an earlier or later time this was accommodated where possible. Participants who were on treatment were advised to call prior to the visit to get advice on which medications to avoid on the morning visit to avoid potential hypoglycaemia. The research doctor or nurse ensured that the participant was satisfied with the information provided on the patient information sheet and the consent form was completed by the participant and countersigned by a member of the research team who had completed appropriate Good Clinical Practice training. Additional clinical risk profiling was performed based on medical history questions required to calculate the QRISK2 score (diagnosis of atrial fibrillation or rheumatoid arthritis, treatment for hypertension and home postal code for the Townsend deprivation score), self-reported smoking status, age, gender and family history of premature coronary artery disease. Anthropometric measurements (height, weight, waist circumference and hip circumference) and BP were taken. Participants were advised to fast for 8 hours prior to the visit for a fasting blood test checking total cholesterol, LDL cholesterol, HDL cholesterol, triglycerides, glucose, hsCRP and renal function (creatinine and estimated glomerular filtration rate eGFR). Based on these measures, the ten-year risk of developing CVD was calculated using the QRISK2 algorithm which is available on their www.qrisk.org website.

Eligibility was confirmed after the first visit once blood results were available to calculate the QRISK2 score. Randomization occurred prior to the second visit. Participants with a QRISK2 ≥ 10% were randomly assigned 1:1 to e-coaching with SOC or SOC alone, stratified according to either “moderate” (QRISK2 between 10 and 20%) or “high” risk (QRISK2 ≥20%).

Randomisation

Randomization was performed using an in-house software tool with concealment of the allocation sequence. Randomization was based on random numbers created in excel 2011 for Mac with 1:1 allocation. 2 sets of four hundred randomised numbers were generated to allow stratification (2 strata). One set was for moderate risk (QRISK2 10-20%) and the other was for high risk (QRISK2 ≥ 20%). Numbers ending with an even digit were allocated to e-coaching; numbers ending with an odd digit were allocated to the SOC treatment arm. The sequence was concealed to researchers involved with recruiting and assessing participants. Randomization into the treatment or control group was performed after confirming eligibility. The randomization sequence and tool were created by RB, who was not involved with the randomization of patients or analysis of the results. The randomization tool was created using PC software, which allocated the treatment group once participant identifier, and their QRISK2 score was entered, to enable stratification (performed by MYK and AB).

Three subsequent predefined visits took place over 6 months; within 2 weeks of screening visit; baseline, 3 months and 6 months from baseline. Email appointment reminders were sent to participants 2 weeks and also 2 days prior to their visit. Assessment was performed using a variety of measures through lifestyle and quality of life questionnaires (EQ-5D-3L, SF-36, recent physical activity questionnaire (RPAQ), blood pressure (BP) checks (Omron 705IT, Omron Corporation, Kyoto, Japan), blood tests (following an 8-hour fast, for lipid profile, glucose, high sensitive C-reactive protein (CRP) and estimated glomerular filtration rate (eGFR)), ultrasound scans (Panasonic CardioHealth System, Panasonic Healthcare Co. Ltd, Yokohama, Japan) oscillometric method to assess pulse wave velocity and pulse wave analysis (Vicorder device, Skidmore Medical, UK).

Baseline visit

All randomised participants received email confirmation of the second visit appointment with instruction of the procedure to be carried out. They were advised to avoid caffeine, alcohol and cigarette smoking prior to the visit for a more accurate PWV measurement.

Prior to the baseline visit all participants were asked to complete a detailed lifestyle questionnaire that would form the basis for lifestyle advice in conjunction with discussion of the results from the screening visit (e.g. BP, blood tests, BMI). The questionnaire assessed recent dietary habits during a typical week, including fruit, vegetable, dairy, meat products including processed meats, daily alcohol intake over the preceding week, questions to gauge psychological, stress and anxiety levels and physical activity questions that tried to elicit duration of moderate, vigorous activities of more than or equal to 10-minute blocks.

During this visit participants were informed of the results of the risk assessment (conforming to information that would normally be available in a primary care setting) and received personalised advice from the research doctor in accordance with guideline recommendations from the NICE and the ESC regarding smoking cessation, weight loss, BP control etc. The personalised lifestyle and risk factor advice was given over 10-15 minutes, based on information from the lifestyle questionnaire and the information available from the screening visit including blood test. Additionally, Vicorder assessment and ultrasound scan (for carotid plaque, CIMT and femoral artery assessment (30 minutes) were performed.

Participants randomised to the e-coaching group were shown how to use the personalised website (10 minutes). Participants were asked to complete additional questionnaires on the day of the visit or at least within 2 weeks of the visit date.

If appropriate, participants were referred to their GP for further tests or interventions (such as initiating BP medication or referral for specialist opinion). We also determined the quality of life (SF-36 and EQ-5D-3L) as well as self-reported physical activity (RPAQ) based on validated questionnaires.

3-month follow-up visit

The 3-month follow-up visit involved repeating most of the assessments from the baseline visit (35 minutes), including blood test, BP measurements, anthropometric measurements (weight, waist and hip circumference), lifestyle questionnaire, and non-invasive measures of vascular function using the Vicorder® device.

6-month follow-up visit

The 6-month follow-up visit involved repeating all assessments from screening and baseline visits for all participants (50 - 60 minutes), including blood test, BP measurements, anthropometric measurements (weight, waist and hip circumference), questionnaires and non-invasive measures of vascular function using the Vicorder device. The CIMT and femoral artery assessment were repeated.

Informed written consent

Participants were asked to complete the consent form once they were satisfied with the aims, methods, anticipated benefits and potential hazards of the study. This information was provided prior to the visit in the form of the PIS. At the visit the researcher also provided a brief summary and clarified any queries or concerns from the participant. The Investigator, or appropriate Good Clinical Practice trained person delegated by the Chief Investigator, obtain written informed consent from each subject prior to any participation/study specific procedures.

Proposed sample size

We based our sample size calculations on using a two-sample t-test with equal variances. The Type I error was set at 5% (two-sided). The inputted standard deviations were based on published inter-study

reproducibility data for our primary end point of PWV: Vicorder measured PWV 0.29 m/s [1]. Aerobic exercise in pre- and stage-1 hypertensive patients for example reduced central PWV after 4 weeks by 1 m/s (12.1 +/- 0.8 m/s to 11.1 +/- 0.8 m/s) [2]. The effect size is likely to be less than what is frequently seen in antihypertensive medication trials after 10-12 months. Sample sizes required for type II errors of 5, 10 and 20% and for four different effect sizes are presented in see Supplementary Table 1. In summary, we proposed a sample size of 200 patients in each treatment arm assuming a dropout rate of 15-20% at the 6-month follow-up visit and having enough power (80%) to detect a small but clinically relevant change in PWV.

Website hosting
The website was hosted in the Netherlands through the HAPPY Globally Foundation. The web team ensured that Dutch and UK legal standards were met to enable secure information transfer. The website team dealt with technical issues arising during the running of the HAPPY London website.

Concurrent medication or treatment
Patients were advised to continue regular medications. The research team initiated no medications. However, in cases where medication was deemed appropriate based on guideline recommendations for cholesterol or BP, a letter of recommendation was sent to their GP. Management was at the discretion of the GP and according to GP’s local protocols.

Assessments

Blood Pressure
BP was taken at screening, 3 months and 6 months. The cuff was deemed an appropriate size for the individual if it covered at least 2/3 of the arm. Systolic and diastolic BP (BP, in mmHg) were measured with a cuff around the left arm after relaxing in a seated position for at least 5 minutes with the left arm rested on table at about the level of the nipple. Legs were uncrossed and the participant was requested not to talk, in accordance with NICE guidelines. At least 2 measures were taken using a fully automated BP monitor (Calibrated Omron 705IT BP). This was repeated if there was a difference of 10mmHg in the systolic or 5mmHg in the diastolic between the two measures. An average of 2 consistent measures was recorded. We did consider including 24-hour blood pressure monitoring but due to limited finances and staffing resources, were unable to include this in the study.

Anthropometric measures
Height (in cm) was measures on bare feet with a portable device (Seca 704s, Hamburg, Germany), which also had a calibrated scale for the body weight (in kg) with the participant in light clothing and without shoes. Height was only measured at the screening visit and along with the serial weight measures. BMI was calculated using the formula - BMI= weight (in kg)/ height^2 (in meters).

Waist circumference (in cm) was taken with a measuring tape at the mid-point between the lower border of the ribs and the upper margin of the pelvis. Hip circumference was measured at the widest observed point of the hip.

Biochemistry
Venesection was performed at the screening visit with a single serum tube of 4-5 mls taken following at least 8 hours of fasting. The serum sample was centrifuged for 30 minutes after the sample was taken and then sent to the lab for analysis.

Blood tubes were labelled with the subjects’ number and date of collection. Samples were analysed in The Doctors Laboratory, 60 Whitfield Street London W1T 4EU. I asked participants to allow long-term storage of samples for future analysis of biomarkers (see consent form in Appendix).

The parameters measured were total cholesterol (mmol/l), HDL (mmol/l), LDL (mmol/l), triglycerides (mmol/l), hsCRP (mg/l), glucose (mmol/l), creatinine (umol/l) and eGFR (mL/min/1.73sqm). The blood tests were performed 3 times in total, with a minimum of 8 hours fast, at the screening visit, 3-months and 6-months.

QRISK2 risk score estimation

The 10-year QRISK2 score was computed using the website www.qrisk.org once blood tests result was available for the screening, 3-month and 6-month visits. This usually occurred on the next working day after the visit. However, during the course of the study its developers updated the QRISK2 algorithm. To ensure I used the most up to date validated QRISK2 algorithm we recalculated scores for all visits at the end of the study. The updated QRISK algorithm scores were used for the final analysis.

Vicorder measurement of pulse wave analysis and pulse wave velocity

Arterial stiffness was determined non-invasively by (i) PWA and (ii) PWV (Vicorder). Performing PWA and PWV measurements took about 15 minutes.

Pulse wave analysis

The research nurse or I performed the PWA and carotid-femoral PWV measurements. The patient was allowed to lie on the bed for about 10-15 minutes before the measurements were taken. The room was temperature controlled and the patient was in a supine position. Patients were specifically advised to refrain from caffeine, alcohol and smoking for at least 8 hours prior to the assessment. The Vicorder measurements were obtained at the baseline visit, 3-months and 6-months follow up visits. PWA was measures first followed by the carotid-femoral PWV.

A brachial BP reading was taken just prior to Vicorder measurements using the same automated Omron machine used for other BP measurements in the study. This BP was used to calibrate the peripheral waveforms obtained from the Vicorder. The Vicorder digitally computed a brachial pressure wave trace with the cuff statically inflated to 70 mmHg using a high-fidelity cuff and volume displacement technique on the left arm in all cases. A brachial-to-aortic transfer function was then applied by the Vicorder software to calculate the waveform and values for central BP [3]. The first and second central systolic peaks were automatically identified by the software and used to calculate the AI (difference in amplitude between first and second systolic peak/pulse pressure x 100). The measurement was repeated 3 times and average of 3 good quality traces was recorded. At least 10 consistent (i.e. no ectopic beats or artefact) beats were required for each PWA measurement.

Pulse wave velocity

Change in carotid-femoral PWV derived from the Vicorder device was the primary end point. We used this surrogate marker, as it appears to have one of the best profiles from the possible surrogate markers available compared to other biomarkers that could be used as potential surrogate markers for CVD[4].

For the assessment of carotid-femoral PWV, cuffs were inflated gently around the thigh and neck, to detect the timing of the waveform between these sites, from which the velocity was calculated. A 10cm wide BP cuff was placed around the upper left thigh to assess the femoral pulse and a 3cm partial cuff was placed around the neck at the level of the left carotid artery. The aortic path length was estimated from the body surface markings according to the instructions from the manufacturers (for the Vicorder device), from the tip of the suprasternal notch to a defined point on the upper part of the femoral cuff (first white stitch mark closest to the groin). The cuffs were simultaneously inflated to 65 mmHg and 2 waveforms of high quality were recorded simultaneously for a target of about 10 consistent beats using a volume displacement method. The foot-to-foot transit time was measured and values for carotid-femoral PWV were automatically obtained from the computer software (supplementary Figure 1).

Carotid intima media thickness

CIMT was determined non-invasively using an ultrasound machine (CardioHealth System, Panasonic). The participant was in a supine position with the head rotated 45° towards the contralateral side of the carotid artery being measured. Automated measure of the CIMT was taken from the posterior artery wall of both carotid arteries with 24 spatial measurements over a 1cm region of interest, 1cm caudal from the flow divider located at the carina of the common carotid artery bifurcation. A vascular probe (Panasonic) was used, with the frequency set at 9 MHz, according to the protocol outlined by the American Society of Echocardiography consensus statement [5]. I assessed for evidence of carotid plaque and measured an automated CIMT. Using the same ultrasound probe, I assessed the femoral arterial bed in the groin to look for evidence of atheroma. The CIMT was automatically calculated once the ultra-sonographer obtained a good quality trace of the posterior wall of the carotid artery. The ultrasound procedure on average took between 5 to 10 minutes.

Intervention

Potential benefits and risks of Electronic Coaching in prevention
Potential advantages of using e-coaching using the Internet and email are that it can be accessed from personal computers or from mobile devices when it is most convenient for the end user, being able to personalize information easily, time saving for the individual and cost-effective for the healthcare system if it reduces need for frequent face-to-face visits.

Challenges surround the use of e-coaching include maintaining cyber security to ensure personal health information is secure. There is a possibility of information overload especially if individuals have numerous suboptimal factors that they need to address and this may even become counter-productive. Adherence to the behavioural program may diminish over time and may limit its effectiveness. Finally, without proven additional effectiveness it would be difficult to implement its widespread use due to the resources and cost associated with maintaining such a program in conjunction with the SOC.

E-coaching
HAPPY London e-coaching website and email reminders
Participants had personal login and passwords. Information was provided as written information on the website with links to other websites and videos. The HAPPY London web-based tool provided each e-coaching group participant with a personalized score for their lifestyle, 10-year CVD risk score and provided tailored advice and information to improve suboptimal factors. Ideal targets were highlighted as goals and the information (lifestyle factors, lifestyle score, risk factors and risk score) were further updated at 3 and 6 months allowing the participant to view their progress, thus providing dynamic tailoring which has been shown to increase efficacy over time [6]. Participants could not ask questions or make comments about specific aspects of their personalized information, in order to test the self-efficacy of the e-coaching tool.

Lifestyle score, as a number and in graph form was provided for each visit to allow participants to view their progress. This included color-coding for easy summary. Lifestyle score of over 8 out of 10 had a green bar, between 6-8 out of 10 an orange bar and less than 6 a red bar. Lifestyle and CVD risk factors were also colour coded for each participant with coloured heart shapes. Green smiley hearts represented optimum factors, orange heart shapes represented factors that were mild to moderately suboptimal and sad red heart shapes represented more than moderately suboptimal factors that required more attention. Participants were advised to log in as often as they wanted. We did not specify a minimum or maximum number of times they were required to log in. We measured the engagement with the platform based on the number of times the participant logged into their account.

Personalized email reminders and tips for lifestyle improvement were sent. Participants with more suboptimal factors received more personalized tips. Bi-weekly health and lifestyle motivational news items with general advice were posted on the website for all participants to view to encourage healthier behaviour. Links to social networks, such as Facebook posting and the ability to allow chosen 'buddies' from family or friends to view their progress was encouraged to further motivate healthy behaviour. Participants received instructions on how to use the website, lasting 5-10 minutes during the baseline visit. The web team developed the content with input from the research team members.

The e-coaching group received an additional 10 minutes (on top of the 10 - 15 minutes discussing results and suggested lifestyle interventions that both groups received). This was to show them how to navigate through the website and personalise some of the features of the web tool and emails. Ideal targets were highlighted as goals and the information (lifestyle factors, lifestyle score, risk factors and risk score) was updated at 3 and 6 months allowing the participant to view their progress, to provide dynamic tailoring which has been shown to increase efficacy over time [6]. Participants were not given the option to interact with the system. They could not ask questions or make comments about specific aspects of their personalised information in order to limit health care resources and test the self-efficacy of the website. Aspects of behavioural change that were addressed included goal setting, social support, review of behaviour goals and the use of prompts.

Control group - Standard of care
This involved face-to-face advice based on the results of the BP, blood test results and lifestyle questionnaire. Factors deemed to be suboptimal were discussed and advice given mainly based on advice that would be obtained during an NHS health check visit (10-15 minutes) and based on

contemporary guidelines [7,8]. If pharmacotherapy was felt to be required, this was communicated to the primary care clinician to prescribe according to local policy.

For those in the control arm or SOC group they did not have access to their information online but information on the result of measurements from the screening visit and personalised advice, based on the lifestyle questionnaire, was the same as that given to the e-coaching group.

Study summary

Methods:
Study design: Randomized controlled trial
Control group: Standard of care. Care given by a physician with guidelines for risk factor control including physical activity targets, BP, blood cholesterol and diet, based on guideline recommendations.

Participants
Study location: London, UK
Population: Asymptomatic, no overt CVD, aged 40-74 with 10-year CVD risk of ≥10% (QRISK2) with Internet access.
Mean age (SD):
Intervention group: 65.1(6.3)
Control group: 65.9(4.8)
Percentage men:
Intervention group: 62% (n=127)
Control group: 64% (n=126)
Number of participants recruited: 402
Participant ethnicity: predominantly white (88%)
Recruited online or offline: Online. However, invitation sent via GP practice data base search meeting QRISK2 score and advertisement through posters including inside London buses.
Intervention

Intervention:
Name of intervention: HAPPY London
Intervention aim: To improve lifestyle and risk factor levels with the addition of e-coaching on top of standard of care.
Intervention features:
The website was personalized for each participant. The website contained tailored information about primary prevention of the modifiable risk factors including blood pressure, physical activity, cholesterol, diet and alcohol. The website also contained links to other websites and videos and twice weekly general health and exercise tips. The website also provided options to nominate a 'buddy' that would also receive emails about the participants goals in order to encourage them and provide motivational support. They also had a link to social networking sites if they wished to share their information. Participants were reminded through personalized automated emails to adhere to the goals and given tips for achieving goals. Compliance with these goals was checked at 3-months and 6-months visits with graphic feedback and progress over time provided. Participants completed questionnaires regarding lifestyle and update of risk factor markers from visits. Feedback of the behaviours was provided at each stage.
How was the intervention introduced to the sample: Face-to-face training session provided lasting about 15 minutes during baseline visit once patient was eligible and randomised?
Was there any contact between the researcher/ health care professional and the sample during the intervention: No formal contact
Duration of the intervention: 6 months

Primary Outcomes:
PWV

Secondary Outcomes:
Augmentation index
LV mass
Carotid IMT
Total cholesterol
HDL cholesterol
LDL cholesterol
Triglyceride
Fasting glucose
CRP
Systolic BP

Diastolic BP
BMI
Waist circumference/Hip circumference
Physical activity – Measurement tool: RPAQ
HRQOL – Measurement tool - EQ5D- We implemented the EQ-5D-3L questionnaire to measurement of QOL

Time points: 3 months and 6 months

Bias Assessment
Random sequence (selection bias) – Computer generated (Excel)
Allocation concealment (selection bias) – Computer program
Blinding of participants and personnel (performance bias) – No blinding
Blinding of outcome assessment (detection bias) – Blinding of outcome measures for analysis
Incomplete outcome data (attrition bias) – Low attrition rate ~6% (similar in both groups. 11 in e-coaching group (5.4%), 13 in the SOC/control group (6.6%), p=0.60 (NS)
Selective reporting (reporting bias) – All primary end points from protocol reported

Detailed Disclosures including ones not relevant to the HAPPY London Study
No financial disclosures declared by any of the authors
JN and LH are the founders of the Happy Globally Foundation. Within the Happy Globally Foundation the e-health tool was developed as a potentially low cost means to improve the lifestyle of many individuals.
MGH has received royalties from Cambridge University Press for textbook ‘’Decision Making in Health and Medicine’’, grants and non-financial support from European Society of Radiology (ESR), non-financial support from European Institute for Biomedical Imaging Research which are all outside the submitted work.
SEP receives consultancy fees from Circle Cardiovascular Imaging Inc., Calgary, Alberta, Canada, which is outside the submitted work.

Rationale and post hoc discussion for 6 months follow-up

A priori we considered 6 months to be the most pragmatic and optimum period. We considered a number of different factors in this decision which included getting the balance correct between allowing enough time for changes to occur in individual participants and also be able to detect differences between groups. Previously published trials of predominantly lifestyle intervention have shown changes in lifestyle and cardiovascular risk factors over 6 months or shorter durations [9,10]. The impact of behavioural change programmes can diminish over a longer period of time and there is risk higher risk of participant dropout that would impact on the ability to detect group differences[11,12]. Although compliance was not the main focus of our study there is a risk that by waiting for longer periods differential effects between groups may no longer be present. We also took into consideration potential resource limitations based on factors including funding, time limited grants and PhD fellow and research staff availability. We considered the various options for follow-up duration and assessed the arguments for and against short, intermediate and longer-term follow-up periods. Overall a pragmatic decision was reached which we felt best reached the balance of the factors that we have outlined above. The decision for 6-month follow-up was therefore decided prior to starting the study and was the basis of funding and ethics approval and had been published on the clinicaltrials.gov website.

Post hoc we also consider that the 6-month period was a good choice. We feel that extending the follow-up period is unlikely to have led to a significant difference in the primary end point of PWV between the two groups given that the 3 months and 6 months results were both similar between the e-coaching and standard of care (SOC) groups. They also did not show any suggestion of divergence between the two groups in the primary end point and in the vast majority of the secondary endpoints. We have now included the 3 months and 6 months comparison graphs of the main variables, including those that are likely to impact directly on the PWV, in the supplementary material (Supplementary Figure 2).

Most of the parameters show convergence or remain parallel, which would suggest that there would be no significant difference with a longer follow-up period. The two main variables where there appears to be divergence of the graphs is in BMI and physical activity levels. For both of these factors in longer term studies most individuals are unable to maintain this level of change and does not necessarily result in differences in biomarkers[13].

We also noted that at baseline the PWV was higher in the SOC group and the improvement was also numerically better in the SOC group (-0.25 ms) compared to the intervention (e-coaching group, -0.16 ms) with a p value of 0.56. The difference remained non-significant even when adjusted for the baseline difference in the PWV between the two groups. Additionally, the trends for PWV reduction between the e-coaching and SOC groups show a convergence of the differences over the 6-month period suggesting that with a prolonged follow-up period the differences are even less likely to be significant.

Our study showed improvements in both the control and the intervention groups to a similar level. This is in part testament to the fact that guideline-based advice and interventions by medical professionals can make modest but significant improvements even in individuals that are considered at risk of future cardiovascular disease many of whom were already pharmacotherapy for primary cardiovascular disease prevention.

The strengths of our RCT include the large number of participants, clear description of the intervention and the control group, the advanced e-coaching tool, low dropout rate at follow-up and thus minimal missing values for the assessment of the primary and secondary end point. We used robust surrogate markers and were able to show improvement in cardiovascular risk factors in both groups to a similar extent based on guideline based primary prevention lifestyle advice and intervention as the standard of care.

In summary, we propose that optimum follow-up period could be controversial but given our a priori rationale and the post hoc analysis with trends showing converging graphs in the primary end point of PWV between the two groups, a longer follow-up would unlikely have given a different outcome. We feel the 6 months follow-up was a pragmatic choice to try and get a balance between likely sustainability of behavioural changes and having enough time for detectable effects to take place.

Questionnaires

SF-36v2® - Short Form 36 version 2 – Health Questionnaire
Ware J, Kosinski M, Bjorner J, Turner-Bowker D, Gandek B, Maruish M. Development. User's Manual for the SF-36v2® Health Survey. Lincoln (RI): QualityMetric Incorporated; 2007.

RPAQ – Recent Physical Activity Questionnaire
http://www.mrc-epid.cam.ac.uk/wp-content/uploads/2014/08/RPAQ.pdf - weblink

EQ-5D-3L – EuroQol 5 dimensions with 3 levels health status measurement questionnaire
https://euroqol.org/wp-content/uploads/2016/09/EQ-5D-3L_UserGuide_2015.pdf - weblink

References

1. Hickson SS, Butlin M, Broad J, Avolio AP, Wilkinson IB, McEniery CM. Validity and repeatability of the Vicorder apparatus: a comparison with the SphygmoCor device. *Hypertens Res* 2009;**32**:1079–1085.

2. Collier SR, Kanaley JA, Carhart R, Frechette V, Tobin MM, Hall AK, Luckenbaugh AN, Fernhall B. Effect of 4 weeks of aerobic or resistance exercise training on arterial stiffness, blood flow and blood pressure in pre- and stage-1 hypertensives. *J Hum Hypertens* 2008;**22**:678–686.

3. O'Rourke MF, Nichols WW. Aortic diameter, aortic stiffness, and wave reflection increase with age and isolated systolic hypertension. *Hypertension* Lippincott Williams & Wilkins; 2005;**45**:652–658.

4. Vlachopoulos C, Xaplanteris P, Aboyans V, Brodmann M, Cifkova R, Cosentino F, De Carlo M, Gallino A, Landmesser U, Laurent S, Lekakis J, Mikhailidis DP, Naka KK, Protogerou AD, Rizzoni D, Schmidt-Trucksäss A, Van Bortel L, Weber T, Yamashina A, Zimlichman R, Boutouyrie P, Cockcroft J, O'Rourke M, Park JB, Schillaci G, Sillesen H, Townsend RR. The role of vascular biomarkers for primary and secondary prevention. A position paper from the European Society of Cardiology Working Group on peripheral circulation: Endorsed by the Association for Research into Arterial Structure and Physiology (ARTERY) Society. *Atherosclerosis* 2015;**241**:507–532.

5. Stein JH, Korcarz CE, Hurst RT, Lonn E, Kendall CB, Mohler ER, Najjar SS, Rembold CM, Post WS. Use of Carotid Ultrasound to Identify Subclinical Vascular Disease and Evaluate Cardiovascular Disease Risk: A Consensus Statement from the American Society of Echocardiography Carotid Intima-Media Thickness Task Force Endorsed by the Society for Vascular Medicine. *Journal of the American Society of Echocardiography* 2008;**21**:93–111.

6. Krebs P, Prochaska JO, Rossi JS. A meta-analysis of computer-tailored interventions for health behavior change. *Prev Med* 2010;**51**:214–221.

7. Perk J, De Backer G, Gohlke H, Graham I, Reiner Ž, Verschuren WMM, Albus C, Benlian P, Boysen G, Cifkova R, Deaton C, Ebrahim S, Fisher M, Germano G, Hobbs R, Hoes A, Karadeniz S, Mezzani A, Prescott E, Ryden L, Scherer M, Syvänne M, Reimer WJMSO, Vrints C, Wood D, Zamorano JL, Zannad F. European Guidelines on cardiovascular disease prevention in clinical practice (version 2012). *Atherosclerosis* Elsevier Ltd; 2012;**223**:1–68.

8. JBS3 Board. Joint British Societies' consensus recommendations for the prevention of cardiovascular disease (JBS3). Heart. 2014. p. ii1–ii67.

9. Yoo HJ, Park MS, Kim TN, Yang SJ, Cho GJ, Hwang TG, Baik SH, Choi DS, Park GH, Choi KM. A Ubiquitous Chronic Disease Care system using cellular phones and the internet. *Diabet Med* 2009;**26**:628–635.

10. Holbrook A, Thabane L, Keshavjee K, Dolovich L, Bernstein B, Chan D, Troyan S, Foster G, Gerstein H, COMPETE II Investigators. Individualized electronic decision support and reminders to improve diabetes care in the community: COMPETE II randomized trial. *CMAJ* 2009;**181**:37–44.

11. Glasgow RE, Kurz D, King D, Dickman JM, Faber AJ, Halterman E, Wooley T, Toobert DJ, Strycker LA, Estabrooks PA, Osuna D, Ritzwoller D. Outcomes of minimal and moderate support versions of an internet-based diabetes self-management support program. *J Gen Intern Med* 2010;**25**:1315–1322.

12. Glasgow RE, Kurz D, King D, Dickman JM, Faber AJ, Halterman E, Woolley T, Toobert DJ, Strycker LA, Estabrooks PA, Osuna D, Ritzwoller D. Twelve-month outcomes of an Internet-based diabetes self-management support program. *Patient Educ Couns* 2012;**87**:81–92.

13. Pietrzak E, Cotea C, Pullman S. Primary and secondary prevention of cardiovascular disease: is there a place for Internet-based interventions? *J Cardiopulm Rehabil Prev* 2014;**34**:303–317.
